# Increased Cystic Fibrosis Transmembrane Conductance Regulators Expression and Decreased Epithelial Sodium Channel Alpha Subunits Expression in Early Abortion: Findings from a Mouse Model and Clinical Cases of Abortion

**DOI:** 10.1371/journal.pone.0099521

**Published:** 2014-06-10

**Authors:** Min Zhou, Jing Fu, Wei Huang, Licong Shen, Li Xiao, Yong Song, Ying Liu

**Affiliations:** 1 Department of Obstetrics and Gynecology, West China Second University Hospital of Sichuan University, Chengdu, People's Republic of China; 2 Sichuan University-The Chinese University of Hong Kong Joint Laboratory for Reproductive Medicine, West China Institute of Women and Children's Health, West China Second University Hospital, Sichuan University, Chengdu, People's Republic of China; University of Texas Health Science Center at Tyler, United States of America

## Abstract

The status of the maternal endometrium is vital in regulating humoral homeostasis and for ensuring embryo implantation. Cystic fibrosis transmembrane conductance regulators (CFTR) and epithelial sodium channel alpha subunits (ENaC-α) play an important role in female reproduction by maintaining humoral and cell homeostasis. However, it is not clear whether the expression levels of CFTR and ENaC-α in the decidual component during early pregnancy are related with early miscarriage. CBA×DBA/2 mouse mating has been widely accepted as a classical model of early miscarriage. The abortion rate associated with this mating was 33.33% in our study. The decidua of abortion-prone CBA female mice (DBA/2 mated) had higher CFTR mRNA and protein expression and lower ENaC-α mRNA and protein expression, compared to normal pregnant CBA mice (BLAB/C mated). Furthermore, increased CFTR expression and decreased ENaC-α expression were observed in the uterine tissue from women with early miscarriage, as compared to those with successful pregnancy. In conclusion, increased CFTR expression and decreased ENaC-α expression in the decidua of early abortion may relate with failure of early pregnancy.

## Introduction

A common pathology of pregnancies is early abortion, and 75% of early abortions are associated with embryo implantation failure [1]. Embryo survival within the maternal uterus is affected by many factors, among which humoral homeostasis in the uterine cavity plays an important role in maintaining cellular homeostasis, which ultimately affects embryo implantation, differentiation and viability [2]. The early embryo after fertilization appears to have a reduced capacity to regulate homeostasis [3]. A large number of identified and postulated molecular mediators are involved in homeostasis regulation in early pregnancy. Humoral homeostasis is maintained via regulation of osmotic gradients, which are largely established by the transport of ions across the epithelium. Various factors are involved in maintaining osmotic gradients; in particular, cystic fibrosis transmembrane conductance regulators (CFTRs) function in cAMP-activated Cl^−^ secretion channels [4] and epithelial sodium channels (ENaCs) mediate the electrogenic influx of Na^+^ across membranes [5]. CFTRs and ENaCs are expressed in the murine female reproductive tract and human uterine epithelia [6], [7]. After mating in mice, decreased CFTR expression and increased ENaC expression are responsible for maximal fluid absorption, which ensures immobilization of the blastocyst and therefore successful implantation [8].

So far, four subunits of ENaC have been cloned in mammals– α, β, γ and δ– and it is known that the α subunit is necessary for channel activity [9], [10]. A recent study showed that ENaC-α activation in the mouse endometrium is maximized at the time of implantation, and it regulates the production and release of prostaglandin E2, which is required for implantation [11]. Moreover, Chen et al. [12] found that CFTR-mediated oviductal HCO_3_^−^ secretion may be vital for early embryo development. These studies suggest the key roles of CFTR and ENaC-α in embryo implantation and maintenance of pregnancy, but the exact mechanisms by which changes in ion channel expression can lead to pregnancy failure are unclear.

Mating between female CBA and male DBA/2 mice is associated with an abortion rate of 20–40% [13]. As the repeatability of the high abortion rate in the peri-implantation stage is reliable in this mating, the CBA×DBA/2 model has been used to investigate the molecular mechanisms and signal pathways underlying early spontaneous abortion [14]–[16]. However, there is no direct evidence of the role of ion channels in this model so far.

Ion channels have been proved vital for reproduction, as previous research had studied ion channels in estrous cycle of mice [6] or in human uteri during the pre-implantation period [8]. However, ion channels expression in decidua after implantation, which potentially indicates what may go wrong at maternal-fetal interface, has not been investigated yet. So it would be interesting to determine whether functional interaction between CFTR and ENaC-α occurs in the decidual tissue of the CBA×DBA/2 mouse abortion model. Therefore, the aim of this study was to investigate the expression of CFTR and ENaC-α in the CBA×DBA/2 model and in clinical cases of early miscarriage in order to determine its potential significance in miscarriage.

## Materials and Methods

### Animals and tissue preparation

This study was carried out in strict accordance with the recommendations of the Guide for the Care and Use of Laboratory Animals of the National Institutes of Health. The protocol was approved by the Ethics Committee of Animal Experiments of Sichuan University. Female CBA and male DBA/2 mice aged 7–8 weeks were purchased from Huafukang Biotech Limited Company (Beijing, China), and male BALB/c mice aged 7–8 weeks were purchased from the animal center of Sichuan University (Sichuan, China). All animals were housed in filter-top cages with autoclaved bedding, autoclaved food and water ad libitum, and a 12 h light/dark cycle. When the mice reached 10 weeks of age, they were mated. CBA×DBA/2 mating represented the abortion model, and CBA×BALB/C mating was used as the control. The appearance of the vaginal plug was defined as day 0.5 of pregnancy. None of the animals received special treatment.

On day 13.5 of pregnancy, the pregnant mice were euthanized by cervical dislocation under sodium pentobarbital-induced anesthesia. The uteri were examined to determine the abortion rate. The abortion-prone embryos were identified by smaller and darker appearance than the viable, pink, healthy embryos, according to the method described in previous reports [16], [17]. The abortion rate was calculated as the number of aborted embryos divided by the total number of embryos (aborted embryos + healthy embryos). After the uterine horns were sectioned longitudinally, the fetal-placental units were peeled from the underlying decidua and discarded, and the decidual tissue was scraped carefully from the uterine muscle. The decidual tissue for histology studies was left on the uterine wall.

### Clinical samples

The investigation with clinical samples was approved by the Medical Research Review Board of West China Second University Hospital of Sichuan University, and written informed consent was obtained from each patient. Between January 2011 and October 2012, 20 women who had a missed abortion were included in the abortion group. Their age was 20–40 years, and the gestational age was 10–12 weeks, as calculated from the last menstrual date and confirmed by ultrasound examination. The diagnosis of missed abortion was based on vaginal bleeding and/or abdominal pain during pregnancy, discontinuation of embryo development, or disappearance of fetal cardiac activity, which was further confirmed by ultrasound examination. Genetic, traumatic, and infective factors that could be responsible for abortions were excluded based on the medical history of the patients and physical and genetic examinations. Twenty-two patients who chose elective abortion within the first trimester of pregnancy were included as controls. They had no history of vaginal bleeding and/or abdominal pain, and had not undergone any hormonal treatment during pregnancy. Moreover, fetal development was normal according to the results of ultrasound examination. The maternal age and gestational age matched those in the abortion group. The decidual tissues of these pregnant women were obtained once the surgery was completed.

### Quantitative RT-PCR

The total RNA from human and mouse decidual tissue was extracted and precipitated. RNA quantification and purification were performed using a NanoVue Plus spectrophotometer (Healthcare Bio-Science AB, Uppsala, Sweden). The nucleotide:protein ratios (A_260_:A_280_) of all the samples were within the acceptable boundaries of 1.9 and 2.1 for both human and mouse samples. Then, cDNA was synthesized using the PrimeScript RT reagent kit (TaKaRa Biotechnology Co. Ltd., Dalian, China). Primers (Sangon Biotech, Shanghai, China) for mouse CFTR, mouse ENaC-α, human CFTR and human ENaC-α cDNA were used, and mouse GAPDH and human ACTIN were used as endogenous controls (Table 1). RT-PCR was performed using SYBR Green real-time PCR Master Mix (Toyobo, Osaka, Japan) on the Applied Biosystems 7900 real-time PCR detection system (ABI, Foster City, CA, USA). The specificity of PCR products was confirmed by analysis of the dissociation curve. Each sample was analyzed in triplicate, and all experiments were repeated three times. Relative gene expression was calculated using the 2^−ΔΔCt^ method.

**Table 1:** Sequences of the primers used for qRT-PCR analysis.

Gene	Forward primer	Reverse primer
Mouse *CFTR*	5′-AGGAGCTTCAACGGTACTGG-3′	5′-GCCTTTGTTAAGGAGGGCTTC-3′
Mouse *ENaC-α*	5′-GCTCAACCTTGACCTAGACCT-3′	5′-GCGGTGGAACTCGATCAGT-3′
Mouse *GAPDH*	5′-TGACCTCAACTACATGGTCTACA-3′	5′-CTTCCCATTCTCGGCCTTG-3′
Human *CFTR*	5′-AGTCCTGGGTATAGAAGTGTGAG-3′	5′-ATTCCAACGGTCGAGTTCTCC-3′
Human *ENaC-α*	5′-CTGGAAGGACTGGAAGAT-3′	5′-GGATGTTGATGTAGTGGAAG-3′
Human *ACTIN*	5′-CTGGAACGGTGAAGGTGACA-3′	5′-AAGGGACTTCCTGTAACAATGCA-3′

### Immunohistochemistry

Human decidua samples and mouse uteri specimens were fixed in formalin, embedded in paraffin, cut to 5- to 7-µm sections, dried, and kept at 4°C in darkness. The sections were then subject to hematoxylin-eosin (HE) staining.

Immunohistochemistry was conducted according to the manufacturer's instructions (Beijing Zhongshan Golden Bridge Biotech Company, Beijing, China). The primary antibodies used for both mouse and human samples were 1∶1,000 rabbit polyclonal anti-CFTR (ACL-006; Alomone Labs, Jerusalem, Israel) and 1∶800 rabbit polyclonal anti-ENaC-α (10924-2-AP; Proteintech Group, Chicago, IL, USA).

### Western blotting

Snap-frozen tissues were homogenized with RIPA lysis buffer and protease inhibitors. The supernatants were solubilized after centrifugation at 12000 rpm for 20 min at 4°C. Following this, equal amounts of protein (50–100 µg) were separated by SDS-PAGE and transferred onto PVDF membranes (Bio-Rad Laboratories Inc., Hercules, CA, USA). The membranes were blocked with 5% milk in Tris-buffered saline (TBS) containing 0.001% Tween-20 (TBST) for 1 h, and incubated in a solution of primary antibodies (CFTR, 1∶1200; ENaC-α, 1∶1000) in 5% milk at 4°C overnight. After washing with TBST, membranes was incubated with HRP-conjugated anti-mouse or anti-rabbit secondary antibodies (1∶10,000), and the signals were visualized with the ECL system (CWBIOTECH, China), using the ChemiDoc XRS system (Bio-rad, Philadelphia, USA) according to the manufacturer's instructions.

### Data analysis

Pearson's χ^2^ test was used to compare the abortion rate and pregnancy rate between groups. Mann-Whitney *U*-test was used to evaluate the implantation rate, mRNA expression level, and protein expression level between groups. Statistical analyses were performed using SPSS version 18.0 (SPSS, Chicago, IL, USA). *P*-values less than 0.05 were considered to indicate statistical significance.

## Results

### CBA×DBA/2 abortion model

**Figure 1:**
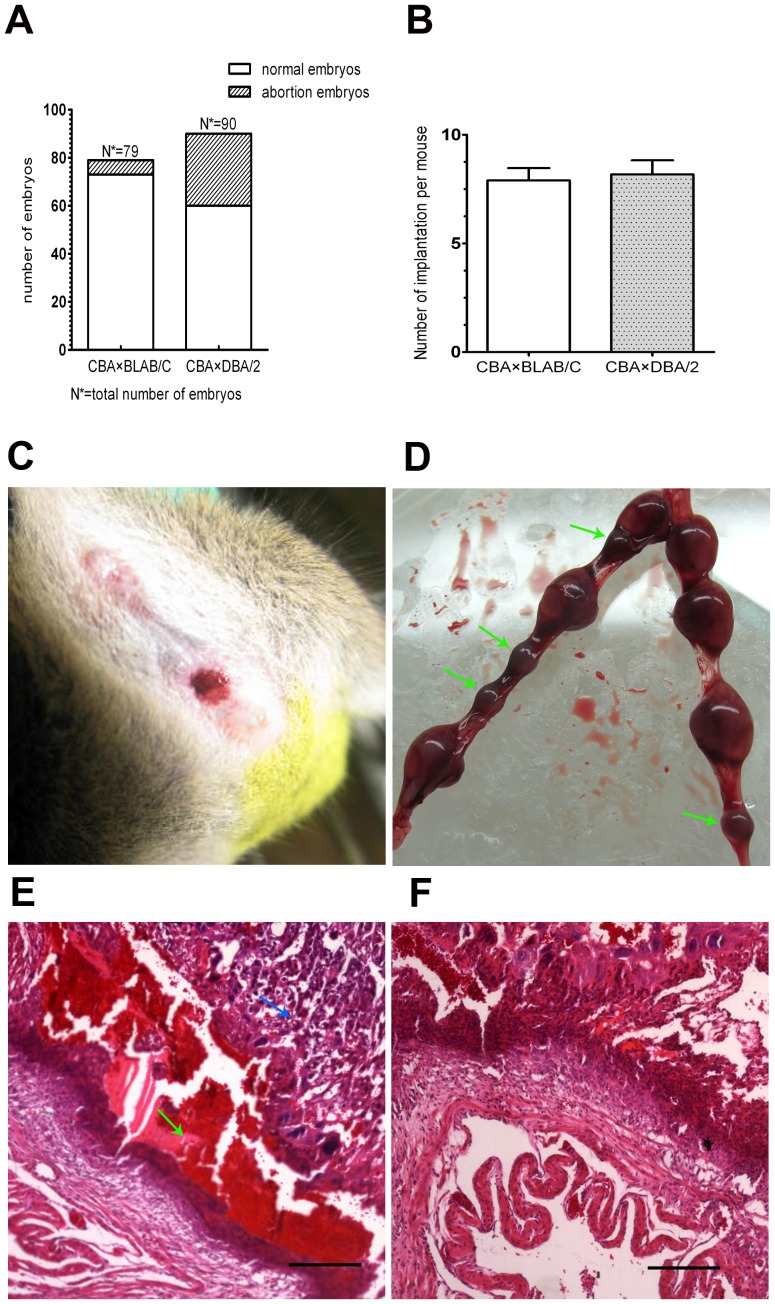
Findings for the CBA×DBA/2 abortion model. (A) In the CBA×DBA/2 group (n = 11), 30 abortion-prone embryos were obtained, while in the control CBA×BALB/C group (n = 10), six abortion-prone embryos were obtained. (B) Implantation rates in the experimental group (8.18±2.14) and control group (7.90±1.79) (P>0.05). (C) Vaginal bleeding was observed on days 7–10 of pregnancy. (D) Representative image of abortion-prone and normal embryos: the arrows indicate the abortion-prone embryos with dysplasia appearance, in which fetuses and placentas can be hardly identified from each other. (E, F) Cross-sections of uterine tissue at the implantation site showed typical ischemia, hemorrhage, and necrosis in abortion-prone embryos (E), but not in healthy embryos (F) (scale bar: 300 µm). Green arrows indicate hemorrhage, and blue arrows indicate necrosis.

The pregnancy rate in the CBA×DBA/2 group was significantly lower than that in the CBA×BALB/C group (66.67% vs. 92.41%, *P*<0.05) (Fig. 1A), while the number of implantation embryos per mouse was comparable (8.18±2.14 versus 7.90±1.79, *P*>0.05) (Fig. 1B).

From day 7 to day 10 of pregnancy, abnormal vaginal bleeding was observed in four of 11 CBA×DBA/2 mice, but in none of the CBA×BALB/C mice (Fig. 1C). In one of the vaginal bleeding CBA mice, these four smaller and darker embryos were confirmed to be abortion-prone embryos based on microscopic findings that showed hemorrhage and necrosis(Fig. 1D, E), compared to healthy embryos(Fig. 1F). The abortion rate (33.33%, 30/90) in the CBA×DBA/2 group was significantly higher than that in the CBA×BALB/C group (7.59%, 6/79) (*P*<0.01) (Fig. 1A).

### Expression of CFTR and ENaC-α mRNA in the decidua

**Figure 2:**
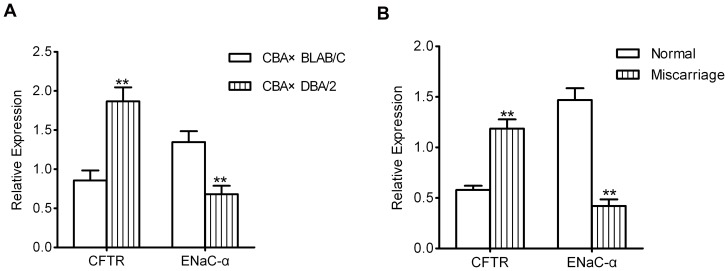
CFTR and ENaC-α mRNA expression in decidua tissue. CFTR mRNA expression increased and ENaC-α mRNA expression decreased in the decidua of CBA×DBA/2 mice, and the difference in expression was statistically significant compared to the CBA×BALB/C mice (A). Similarly, higher CFTR mRNA expression and lower ENaC-α mRNA expression were detected in the decidua of women who underwent abortion (B). **P<0.01.

Quantitative RT-PCR revealed increased CFTR mRNA (0.86±0.13 vs. 1.87±0.18) and decreased ENaC-α expression (1.35±0.14 vs. 0.68±0.11) in the decidua of CBA×DBA/2 mice compared to CBA×BALB/C mice (*P*<0.01). Similarly, increased CFTR and decreased ENaC-α mRNA expression was found in the human decidua samples of miscarriage compared to decidua samples from normal pregnancies (0.58±0.04 vs. 1.19±0.09 and 1.47±0.12 vs. 0.42±0.07 respectively; *P*<0.01). Therefore, both sets of data showed upregulation of CFTR mRNA expression and downregulation of ENaC-α mRNA expression in abortion-prone decidua tissue (Fig. 2).

### Expression and distribution of CFTR and ENaC-α protein in the decidua

**Figure 3:**
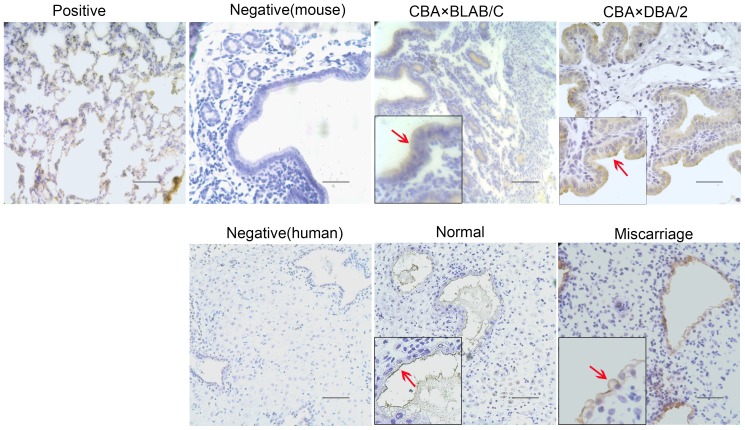
Immunohistochemical analysis of CFTR expression in mouse and human tissue samples. CFTR protein is indicated by arrows in the image. Representative images (400× for the insets) are shown. CFTR was detected as brown signals located mainly to the cell membrane. The expression of CFTR protein was stronger in the decidua of CBA×DBA/2 mice than CBA×BALB/C mice; moreover, it was stronger in women who had a miscarriage than normal pregnant women. Rat lung tissue was used as the positive control. Negative controls were obtained by replacing the first antibody with PBS. (scale bar: 50 µm).

**Figure 4:**
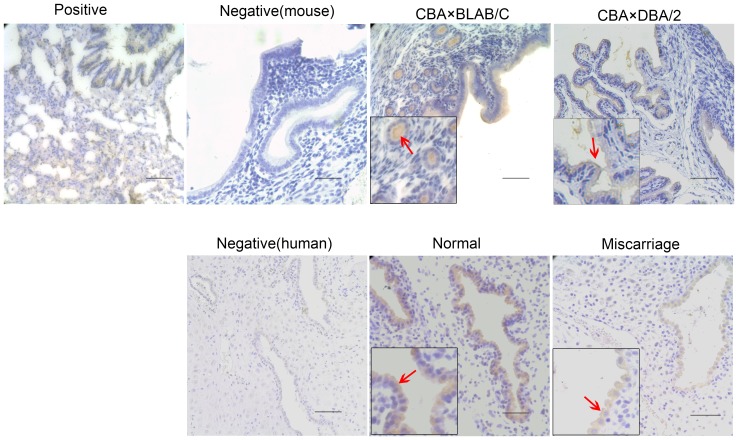
Immunohistochemical analysis of ENaC-α staining in mouse and human tissue samples. ENaC-α is indicated by arrows in the image. Representative images (400× for the insets) are shown. ENaC-α was detected as brown signals located mainly to the cell membrane. The expression of ENaC-α protein was weaker in the decidua of the CBA×DBA/2 mice compared to the CBA×BALB/C mice; moreover, it was weaker in the women who underwent miscarriage than normal pregnant women. Rat lung tissue was used as the positive control. Negative controls were obtained by replacing the first antibody with PBS (scale bar: 50 µm).

As shown in Figure 3 and Figure 4, in mice, CFTR and ENaC-α were predominantly detected in the membrane of glandular and luminal epithelial cells of the decidua, but were almost absent in stromal cells. However, in human decidual tissue, CFTR and ENaC-α proteins were detected notably on the membrane of glandular cells. Compared to normal decidua samples from mice and humans, CFTR expression was higher (Fig. 3) and ENaC-α expression was weaker (Fig. 4) in the mouse abortion-prone and human miscarriage samples.

**Figure 5:**
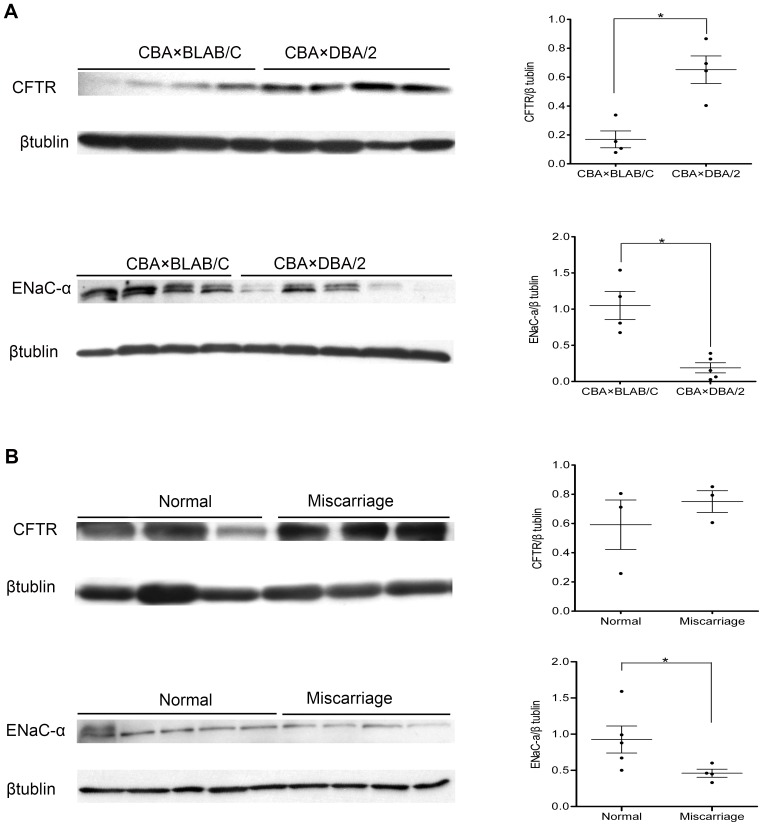
CFTR and ENaC-α protein detection by western blot analysis. The expression of CFTR protein was stronger and the expression of ENaC-α protein was weaker in the decidua of CBA×DBA/2 mice compared to CBA×BALB/C mice (A). Moreover, the expression of CFTR protein was stronger and the expression of ENaC-α protein was weaker in the decidua of women who underwent a miscarriage than normal pregnant women (B). β-tubulin was used as the internal control (approximately 43 kD). Representative images are shown. Densitometry analysis data for CFTR and ENaC-α expression are normalized to the control (100%) for comparison. *P<0.05.

From the results of Western blot analysis, CFTR was represented as a mature fully glycosylated band (approximately 170 kD) and ENaC-α protein was represented as an 85 kD band (approximately), as expected. Densitometry analysis was used for analyze CFTR and ENaC-α expression. Overexpression of CFTR protein and decreased expression of ENaC-α protein was detected in the abortion-prone CBA×DBA/2 decidua tissues (*P*<0.05) (Fig 5A). Similarly, ENaC-α protein expression in human miscarriage samples was lower than that in normal pregnancy samples (*P*<0.05), while CFTR protein expression was higher (*P*>0.05) (Fig. 5B).

## Discussion

The interaction between CFTR and ENaC has been proposed as the major mechanism regulating fluid absorption and secretion in the endometrial epithelia [6]. It is considered that downregulated CFTR and upregulated ENaC in the endometrial epithelia of mice are required to achieve maximal fluid absorption necessary for successful implantation [8]. The balance of these ion channels may be important in the cellular microenvironment during early pregnancy. In our study, the results demonstrate for the first time the differential expression of CFTR and ENaC-α between the decidual tissue of abortion-prone and normal pregnant mice. Overexpression of CFTR and inadequate expression of ENaC-α was observed in the decidua from abortion-prone mice and women who had a miscarriage. CFTR have shown to be a negative regulator of ENaC in mouse endometrial epithelium which is important for liquid microenvironment during peri-implantation [18]. The abnormal expression of CFTR and ENaC-α might disrupt the cellular microenvironment that is harmful for embryo implantation or maintain of pregnancy; or might result from degeneration or necrosis of decidua as a consequence of miscarriage. However, the up-regulation of CFTR and down-regulation of ENaC-α in mechanism of miscarriage remains unclear.

CFTR and ENaC-α are ion channels that are co-expressed in a variety of cell types, including human endometrial and mouse endometrial cells. A previous study conducted in mice during pre-implantation suggested that the CFTR protein was predominantly found in stromal cells, but not in epithelial cells (neither luminal nor glandular), while the ENaC protein was predominantly localized to both luminal and glandular epithelial cells [8]. Interestingly, in our study, we observed that the location of CFTR and ENaC-α protein expression in mouse uteri was not the same as that in human uteri. There are several reasons that might lead to these inconsistencies. Firstly, polyclonal antibodies used in the study might result in non-specific expression; Secondly, the human decidua samples were obtained from cases of missed abortion and not fresh inevitable abortion; therefore, the tissue morphology might have been abnormal and luminal epithelia may not have been present.

The CBA×DBA/2 mouse abortion model was used to investigate the role of CFTR and ENaC-α in early pregnancy. Consistent with previous reports, we found a high prevalence of abortion with this mating pair. The exact mechanism associated with the spontaneous abortion in this model is unclear, and most studies have focused on abnormal immune reactions at the maternal-fetal interface [13], [15]–[16]. Several studies have demonstrated the predominance of Th_2_-type cytokines over Th_1_-type cytokines at the maternal-fetal interface in cases of successful pregnancy [19]–[21]. However, in the CBA×DBA/2 model, increased expression of Th_1_-type cytokines such as interleukin (IL)-1, interferon (IFN)-γ, and tumor necrosis factor (TNF)-α, and decreased expression of Th_2_-type cytokines such as transforming growth factor (TGF)-β2, IL-4, and IL-10 are responsible for the spontaneous abortion [15], [22]–[24]. Interestingly, significantly higher IL-1β levels were found in the uterine fluid from women with repeated pregnancy failure after in vitro fertilization/embryo transfer [25].

The interaction between ion channel and Th1-type cytokines is unclear. Some studies on respiratory and gastrointestinal system have suggested that IL-1β specifically upregulated CFTR gene expression in human intestinal T84 cells [26]–[27]. Previous studies revealed that cytokines such as INF-γ and TNF-α regulate the activity of CFTR and ENaC-α in many kinds of primary cultured cell; although the mechanism is still unknown and controversial [28]–[31]. Therefore, cytokines INF-γ and TNF-α should be responsible for altered expression of CFTR and ENaC-α in decidua of miscarriage. We speculate that, in the decidual tissue from abortion-prone CBA mice, Th_1_-type cytokines might effects on Na^+^ absorption and Cl^−^ secretion via alteration of the expression of CFTR and ENaC. Increased Cl^−^ secretion and decreased Na^+^ absorption lead to detrimental excessive fluid accumulation within the uterine cavity [32]–[34], and finally adversely influence implantation and embryo growth, leading to abortion. However, the exact mechanism of CFTR and ENaC in early abortion involves Th_1_-type cytokines need further investigation.

One of the limitations of our study is that we did not use fresh abortion tissue from cases of miscarriage, and therefore, the decidual tissue may not truly represent in vivo tissue in human inevitable abortion. Thus, the precise expression levels of CFTR and ENaC-α in human miscarriage remain to be explored.

In conclusion, our current results show increased CFTR and decreased ENaC-α expression in the decidua from mice or human abortions. The expression of ion channels are results or causes of miscarriage remains unknown. The exact mechanism involving ion channel in miscarriage warrant further study.
